# Crystal induced arthropathies—a comparative study of 40 patients with apatite rheumatism, chondrocalcinosis and primary synovial chondromatosis

**DOI:** 10.3389/pore.2024.1611454

**Published:** 2024-03-05

**Authors:** Miklós Bély, Ágnes Apáthy

**Affiliations:** ^1^ Department of Pathology, Hospital of the Order of the Brothers of Saint John of God in Budapest, Budapest, Hungary; ^2^ Department of Rheumatology, St. Margaret Clinic, Budapest, Hungary

**Keywords:** apatite rheumatism, chondrocalcinosis, primary synovial chondromatosis, conventional stains, non-staining technique

## Abstract

**Introduction:** Apatite rheumatism (AR), chondrocalcinosis (Ch-C), and primary synovial chondromatosis (prSynCh) are regarded as distinct clinical entities. The introduction of the non-staining technique by Bély and Apáthy (2013) opened a new era in the microscopic diagnosis of crystal induced diseases, allowing the analysis of MSU (monosodium urate monohydrate) HA (calcium hydroxyapatite), CPPD (calcium pyrophosphate dihydrate) crystals, cholesterol, crystalline liquid lipid droplets, and other crystals in unstained sections of conventionally proceeded (aqueous formaldehyde fixed, paraffin-embedded) tissue samples. The aim of this study was to describe the characteristic histology of crystal deposits in AR, Ch-C, and prSynCh with traditional stains and histochemical reactions comparing with unstained tissue sections according to Bély and Apáthy (2013).

**Patients and methods:** Tissue samples of 4 with apatite rheumatism (Milwaukee syndrome), 16 with chondrocalcinosis, and 20 with clinically diagnosed primary synovial chondromatosis were analyzed.

**Results and conclusion:** Apatite rheumatism, chondrocalcinosis, and primary synovial chondromatosis are related metabolic disorders with HA and CPPD depositions. The authors assume that AR and Ch-C are different stages of the same metabolic disorder, which differ from prSynCh in amorphous mineral production, furthermore in the production of chondroid, osteoid and/or bone. prSynCh is a defective variant of HA and CPPD induced metabolic disorders with reduced mineralization capabilities, where the deficient mineralization is replaced by chondroid and/or bone formation. The non-staining technique of Bély and Apáthy proved to be a much more effective method for the demonstration of crystals in metabolic diseases than conventional stains and histochemical reactions.

## Introduction

### Serendipity—the unexpected and incidental discovery

Legs amputated because of Mönckeberg’s sclerosis and/or atherosclerosis were studied by the authors [1, 2]. They observed unstained tissue sections of the conventionally processed surgical specimens viewed with polarized light; variable amounts of cholesterol (CC—[C_27_H_46_O]), crystalline liquid lipid droplets (CL), calcium hydroxyapatite (HA—[Ca_5_(PO_4_)_3_(OH)]) and calcium pyrophosphate dihydrate (CPPD—[Ca_2_P_2_O_7_.2H_2_O]) crystals were detectable.

It is generally accepted that CC and CL cannot be detect in conventionally processed tissue samples stained with HE or other aqueous dyes; only empty spaces indicate the previous presence of cholesterol and crystalline lipids [3].

Lipid complexes can exceptionally remain in traditionally processed HE-stained sections, if these complexes did not dissolve in conventional fat solvents (ethyl alcohol, acetone, xylene, chloroform, methanol) or in aqueous dye solutions.

In HE stained sections of traditionally processed surgical specimens the small and soluble, weakly birefringent HA crystals are not visible with a conventional light microscope [4] or under polarized light [5, 6]. Some authors believe that HA crystals are identical with the amorphous mineral deposits stained with Alizarin red S [6–10], although Alizarin red S staining is not specific for HA crystals [11]. Moreover, calcium and phosphorus in crystalline structure do not stain with Alizarin red S or with the von Kossa reaction [12–16].

Using a professional polarizing microscope with high brightness (at least 100-Watt illumination) the weak birefringent HA crystals can be detected exceptionally in HE stained sections viewed under polarized light [15, 16], while the large and less soluble CPPD crystals with a relatively strong birefringence are more often demonstrated, and are usually well identifiable [15–17].

In contrast to the classical staining methods the unstained technique is much more effective in the detection of crystals and crystalline structures of various metabolic disorders [1, 12–16].

### Objective

The authors wished to ascertain with their sensitive non-staining technique the presence of various crystals in conventionally processed tissue samples of patients with the clinical diagnosis of apatite rheumatism (AR), chondrocalcinosis (Ch-C), and primary synovial chondromatosis (prSynCh).

## Patients and methods

Between 1985 and 2010 surgical specimens of 101,855 patients were processed in the Department of Pathology of the National Institute of Rheumatology (ORFI) and of the Hospital of the Order of Brothers of Saint John of God (BIK).

Among these, apatite rheumatism (Milwaukee syndrome) was diagnosed clinically in 4 (0.0039%), chondrocalcinosis in 16 (0.016%), and primary synovial chondromatosis in 20 (0.020%) patients.

Sixteen (16) paraffin embedded tissue blocks of the 4 patients with apatite rheumatism, 40 of the 16 patients with chondrocalcinosis, and 37 blocks of the 20 patients with prSynCh were available.

The tissue samples were fixed in an 8% aqueous solution of formaldehyde at pH 7.6 for at least 24 h at room temperature (20°C) and embedded in paraffin.

Serial sections were examined without staining [1, 12–16], with HE staining [18], as well as with special stains recommended in the literature, and were examined with the light microscope and under polarized light, respectively.

Amorphous calcium phosphate [Ca_3_(PO_4_)_2_] and/or calcium carbonate [CaCO3] deposits—characteristically accompanying HA and CPPD crystal deposits—were identified with Alizarin red S staining (specific for calcium) [19, 20] and the von Kossa reaction (specific for phosphate and/or carbonate) [19, 21].

Conventionally stained tissue sections were compared with unstained sections according to Bély and Apáthy (2013) (description of non-staining method see as Appendix at the end of the manuscript).

The amount of amorphous mineral deposits, chondroid, osteoid and/or bone formations were assessed by conventional stains and reactions using a semiquantitative score system: “0”—no mineral deposits, chondroid and/or bone formation, “1”—minimal mineral deposits, chondroid and/or bone formation, “2”—moderate mineral deposits, chondroid and/or bone formation, “3”—abundant (massive) mineral deposits, chondroid and/or bone formation [15, 16]. The differences were calculated and compared with the student (Welch) T-probe [22].

Demographics of the patients with the clinical diagnosis of AR, Ch-C and prSynCh were compared with the Student (Welch) T-probe [22].

The effectivity of non-staining technique was characterized with Pearson’s chi-squared (χ^2^) test comparing the prevalence of deposited crystals in unstained tissue sections with HE [22]. The difference between two cohorts of samples was regarded “significant” at an alpha level of 0.05.

Standard and unstained sections were examined with a professional high-brightness (100-Watt) microscope (Olympus BX51); in selected cases, electron microscopy and electron diffraction were also performed (JEM 100CX).

## Results

### Demographics of patients with clinically diagnosed apatite rheumatism, chondrocalcinosis and primary synovial chondromatosis

The mean age of patients with the clinical diagnosis of prSynCh (50.20 years) was low at the time of surgery.

The mean age of patients with AR was high at the time of surgery compared to the patients with the clinical diagnosis of prSynCh (74.0 years versus 50.20 years; *p* < 0.0001) (Tables 1, 2).

**TABLE 1:** Sex, mean age with SD and range (in years) of 40 patients with clinically diagnosed G, AR, Ch-C or prSynCh.

Clinical diagnosis	Number of patients (tissue samples)	Mean age in years at surgery ± SD	Range (In years)
Apatite rheumatism (HA)	4 (16)	74.00 ± 7.70	66–82
Female	3 (12)	76.67 ± 6.81	69–82
Male	1 (4)	66.0	66.0
Chondrocalcinosis (CPPD)	16 (40)	63.67 ± 21.17	39–81
Female	14 (34)	62.08 ± 14.02	39–81
Male	2 (6)	74.00 ± 1.41	73–75
Primary synovial chondromatosis	20 (37)	50.20 ± 12.51	30–76
Female	13 (22)	53.15 ± 10.49	40–74
Male	7 (15)	44.71 ± 14.90	30–76

Combined (co-existent) diseases did not occur in our patient cohorts with gout, apatite rheumatism, chondrocalcinosis, and primary synovial chondromatosis.

**TABLE 2:** Level of significance (“p” value < 0.05) comparing the mean age of 40 patients with clinically diagnosed AR, Ch-C or prSynCh.

Clinical diagnosis number of patients	Mean age of patients	*p* < 0.05	Mean age of females	*p* < 0.05	Mean age of males	*p* < 0.05
AR *n* = 4 vs. Ch-C *n* = 16	74.00 vs. 63.67	0.8440	76.67 vs. 62.08	0.0361	66.00 vs. 74.00	—
AR *n* = 4 vs. prSynCh *n* = 20	74.00 vs. 50.20	0.0018	76.67 vs. 53.15	0.0061	66.00 vs. 44.71	—
Ch-C *n* = 16 vs. prSynCh *n* = 20	63.67 vs. 50.20	0.0074	62.08 vs. 53.15	0.0920	74.00 vs. 44.71	0.0018

Differences were not calculated between male patients with the clinical diagnosis of AR and Ch-C, because of a zero divisor.

There was no significant difference between patient cohorts with AR and Ch-C (74.0 years versus 63.67 years; *p* < 0.0840), except the women (76.67 years versus 62.028 years; *p* < 0.0361).

Table 1 summarizes the demographics of patient cohorts with the clinical diagnosis of AR (*n* = 4), Ch-C (*n* = 16) or prSynCh (*n* = 20).

Table 2 summarizes the “p” values of significance (at an alpha level of 0.05) comparing the mean age of patient cohorts with the clinical diagnosis of AR (*n* = 4), Ch-C (*n* = 16) or prSynCh (*n* = 20).

### Microscopic characteristics of apatite rheumatism, chondrocalcinosis and primary synovial chondromatosis

#### Microscopic characteristics of HA (hydroxyapatite [Ca_5_(PO_4_)_3_(OH)]) crystal deposits in patients with clinically diagnosed apatite rheumatism

Apatite rheumatism was characterized histologically by intra- or periarticular accumulation of HA [Ca_5_(PO_4_)_3_(OH)] crystals (with or without CPPD), typically in association with amorphous calcium phosphate [Ca_3_(PO_4_)_2_], and/or calcium carbonate [CaCO_3_] deposits of irregular shape (Figures 2A–D).

According to our semi-objective score system, in AR the mineral deposition was 1.063 per tissue sections, accompanied with minimal chondroid formation (0.063 per tissue sections); osteoid or newly formed bone tissue was not detected [23].

The nearly complete absence of inflammation (with or without adjacent fibrosis) was characteristic for amorphous mineral deposits associated with HA [11, 14, 15, 24, 25].

The individual HA crystals in the synovial membrane caused acute inflammation, and provoked phagocytosis of neutrophilic leukocytes in all cases [24, 25].

Acute inflammation of joints was accompanied with severe clinical symptoms: sudden onset of severe pain, synovial fluid effusion, swelling, tenderness, restricted motion, joint destruction of rapid progression, deformity and instability of joints [23–25].

In unstained sections, using a professional polarizing microscope with high, at least 100-Watt brightness, the clusters (1–5 µm) of HA crystals were visible, and were birefringent under polarized light (×100) [13, 14]. The clusters formed aggregates of 100 µm or larger, which were visible with an objective of ×40 with non-staining techniques [13, 14].

The birefringence of HA prisms was weakly positive (δ = 0.007) compared to the stronger positive birefringence of CPPD crystals (δ = 0.017); the large and strongly birefringent CPPD crystals dominated deceptively the microscopic fields, in cases of coexisting HA and CPPD crystals at 60-Watt illumination, or using a Red I compensator (the Red I compensator notably reduces the intensity of illumination) [13, 14].

In our previous study we examined 16 tissue samples of 4 patients with clinically diagnosed apatite rheumatism; 9 joints were operated: 5 knees, 3 shoulders and 1 hip.

In traditionally fixed and processed tissue sections stained with HE, Alizarin red S or with the von Kossa reaction, HA crystals were not found.

With non-staining techniques HA crystals were present in all 4 patients, and were detected in 10 (62.5%) of the 16 unstained sections (with or without CPPD crystals) [16].

In HE stained tissue section cholesterol crystals (CC—[C_27_H_46_O]) were not found, but in unstained sections CC crystals were detected in 3 of 4 patients [23].

**FIGURE 1:**
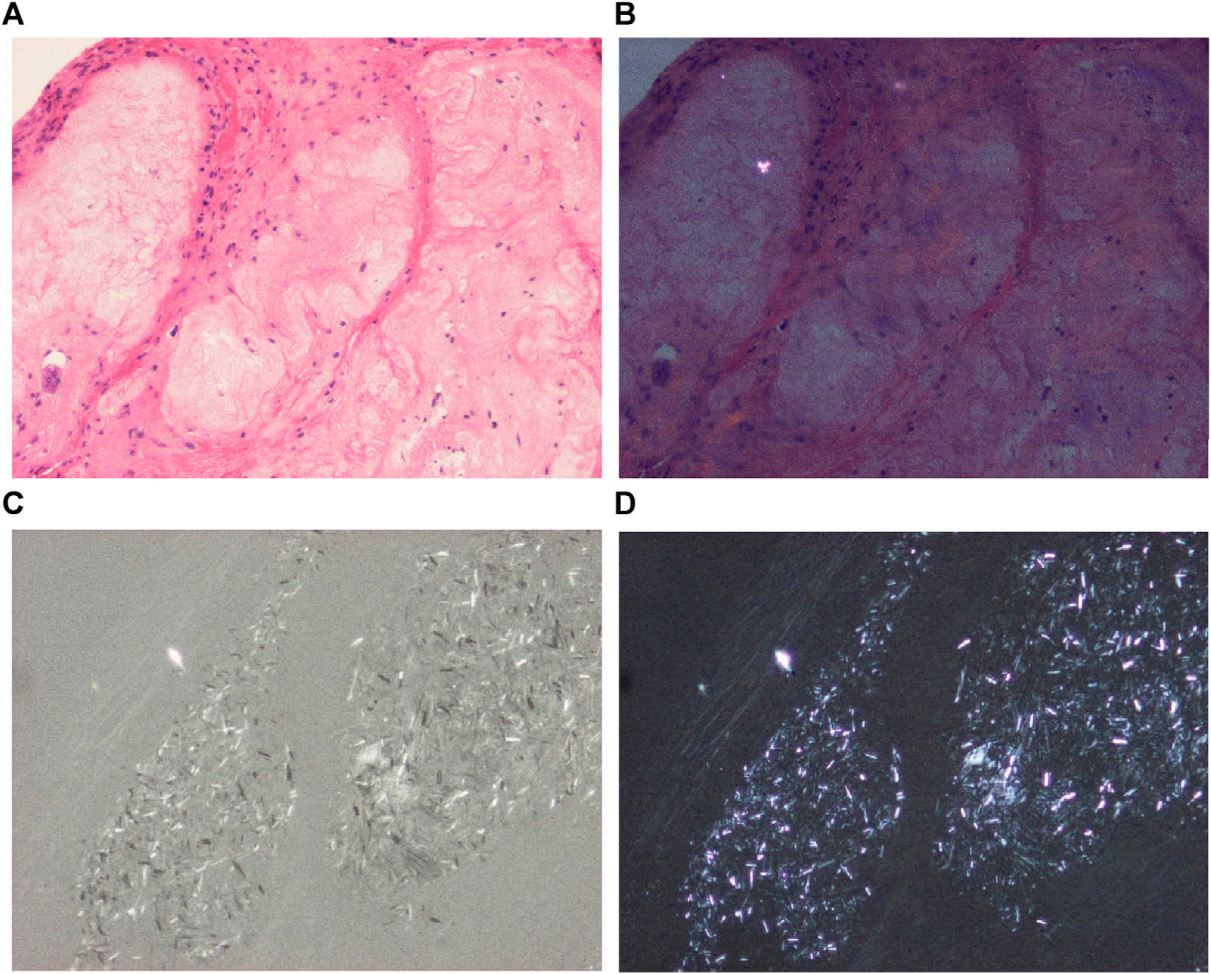
Hydroxyapatite arthropathy (Milwaukee syndrome, apatite rheumatism) induced by hydroxyapatite (HA) crystals, viewed with the light microscope and under polarized light, respectively. The weakly birefringent small HA crystals are readily soluble and are not detected in conventionally fixed tissue specimens stained with HE. **(A)** HE, viewed with the light microscope, ×100, **(B)** HE, viewed under polarized light (birefringent particle is a remnant of paraffin), same as **(A)** ×100. Mixed HA, CPPD, and unidentified needle-shaped crystals at high brightness illumination (100-Watt); less illumination (60-Watt) deceptively highlights the dominant (more birefringent) crystals. **(C)** Unstained section, viewed under polarized light with high brightness illumination (100-Watt), same microscopic field as **(A)** ×100, **(D)** same as **(C)** with 60-Watt illumination.

**FIGURE 2:**
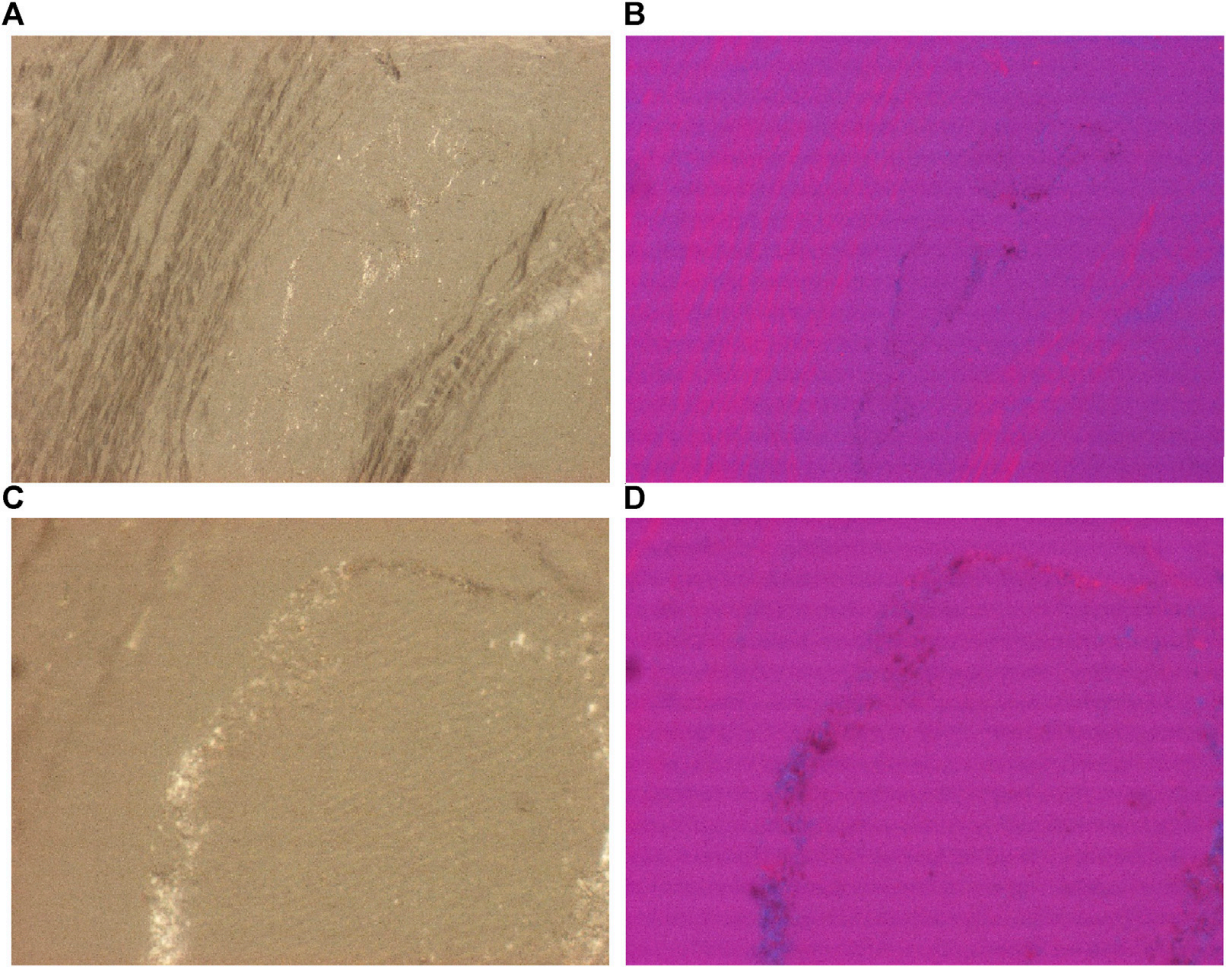
Prepatellar bursa, hydroxyapatite arthropathy, HA crystal prisms, unstained section, viewed under polarized light. HA crystals are colorless, translucent fragments or small (50–500 nm) rod-shaped prisms. Under polarized light with Red I. compensator the birefringence of **HA** crystals is weak and positive. **(A)** HA crystals, unstained sections, viewed under polarized light, ×100, **(C)** same as **(A)** ×600. **(B)** HA crystals, unstained sections, viewed under polarized light, using Red I compensator, ×100, **(D)** same as **(B)** ×600.

The characteristics of HA crystal deposits are demonstrated in Figures 1, 2.

#### Microscopic characteristics of CPPD (calcium pyrophosphate dihydrate [Ca_2_P_2_O_7_.2H_2_O]) crystal deposits in patients with clinically diagnosed chondrocalcinosis

CPPD crystal deposition was accompanied with more or less amorphous calcium phosphate [Ca_3_(PO_4_)_2_] and/or calcium carbonate [CaCO_3_] deposits of irregular shape [16, 24, 25].

Based on our semi-objective score system in Ch-C the mineral deposition was 1.054 per tissue sections, accompanied by minimal chondroid formation (0.054 per tissue sections); osteoid or newly formed bone tissue was not detected [23].

In synovium, menisci or hyaline cartilage, etc. the CPPD crystals were often asymptomatic, the inflammatory reaction around mineral deposits were usually moderate or absent.

Occasionally CPPD crystals provoked a cellular reaction (with or without macrophages) and also caused joint damage; the affected joints were usually swollen, warm, and severely painful [24].

CPPD crystals were less soluble in an 8% aqueous formaldehyde solution or water containing dyes than HA crystals; in tissue sections stained with HE, Alizarin red S or with the von Kossa reaction, occasionally some CPPD crystals remained back and were demonstrable with polarized light.

Large amounts of amorphous mineral deposits obscured CPPD crystals, but the crystals not stained with Alizarin red S or with the von Kossa reaction [14].

**FIGURE 3:**
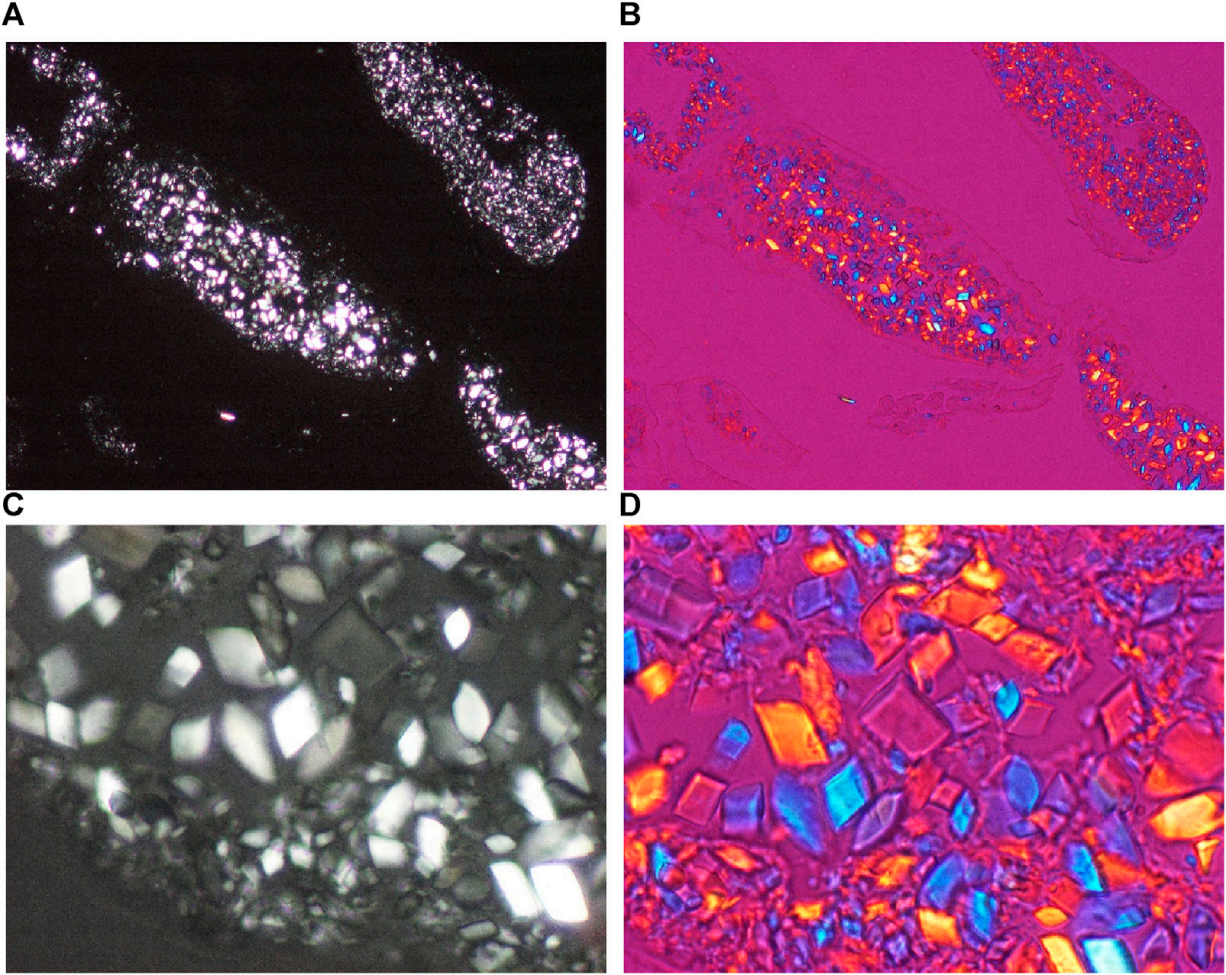
Chondrocalcinosis, knee joint, synovial membrane, unstained section, viewed under polarized light. CPPD plane crystals are of hexagonal, rhomboid, trapezoid, parallelogram-shape or fragments of these, they range in size from submicroscopic to 40 μm, and show a relatively strong positive birefringence according to the long axis of the crystals with Red I. compensator. **(A)** CPPD crystals, unstained sections, viewed under polarized light, ×100, **(C)** ×600. **(B)** CPPD crystals, unstained sections, viewed under polarized light, using Red I compensator, same as **(A)**, ×100, **(D)** ×600.

CPPD typically showed plane crystals of hexagonal, rhomboid, trapezoid, parallelogram-shape or fragments of these (Figures 3A–D).

With Red I compensator the CPPD crystals showed a relative strong positive birefringence (δ = 0.017), in contrast to the weak positive birefringence of HA crystals (δ = 0.007).

In our previous study we examined 40 tissue samples of 16 patients with clinically diagnosed chondrocalcinosis; 16 joints were operated: 8 knees, 4 hips, 2 wrists, 1 shoulder, and 1 elbow.

In traditionally fixed and processed tissue sections stained with HE, Alizarin red S or with the von Kossa reaction, CPPD crystals were found in 5 of 16 (31.25%) patients, and were detected in 8 (20.0%) of the 40 tissue sections.

With non-staining techniques CPPD crystals were present in all 16 patients, and were detected in 22 (55.0%) of the 40 unstained sections (with or without HA crystals) [16, 24, 25].

In HE stained tissue section cholesterol crystals (CC—[C_27_H_46_O]) were not found, and in unstained sections CC crystals were detected in 11 of 16 patients [23].

Figures 3A–D demonstrates characteristic CPPD crystal deposits in a patient with clinically diagnosed chondrocalcinosis.

#### Microscopic characteristics of HA and/or CPPD crystal deposits in patients with clinically diagnosed primary synovial chondromatosis

Primary synovial chondromatosis (prSynCh) was characterized clinically by slow progression of symptoms: joint pain, and swelling, stiffness, limited motion of the affected joint, intraarticular fluid, tenderness and creaking, grinding, or popping noise during movement (crepitus) or locking of the joint.

Abundant chondroid and/or bone formation (with or without true medullary spaces) was usually accompanied only with minimal amorphous calcium phosphate [Ca_3_(PO_4_)_2_] and/or calcium carbonate [CaCO_3_] deposition [15, 16].

Calculated by our semi-objective score system in prSynCh the average chondroid and/or bone formation was 2.135 per tissue sections, accompanied with minimal mineral deposition (0.270 per tissue sections) [23].

In our previous study we examined 37 tissue samples of 17 patients with clinically diagnosed prSynCh; 18 joints were operated: 12 knees, 4 hips, 2 elbows.

In traditionally fixed and processed tissue sections stained with hematoxylin eosin HA crystals were not detected, CPPD crystals were found in 4 of 17 (23.53%) patients, and in 4 of 37 (10.81%) tissue sections.

With the non-staining technique only 17 tissue sections of 8 patients were examined; HA and CPPD crystals were present in all patients and in 12 of 17 tissue sections [15, 16].

In HE stained tissue sections of prSynCh no cholesterol crystals (CC—[C_27_H_46_O]) were not found, but in unstained sections CC crystals were detected in 2 of 8 patients [23].

**FIGURE 4:**
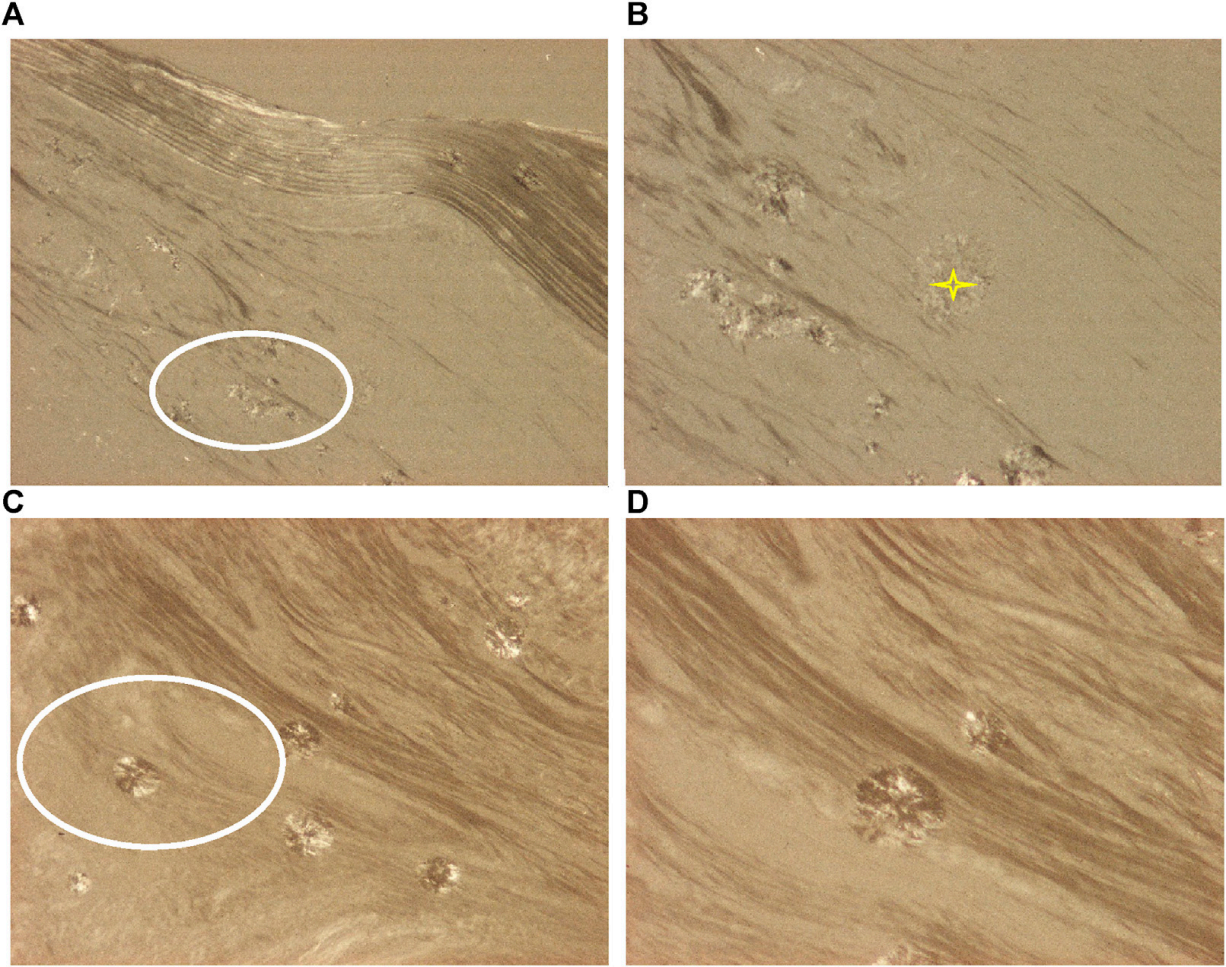
Synovial osteo-chondromatosis, knee joint, HA crystal prisms and clusters. The small prisms of HA crystals (white ellipses) are arranged in larger spheroid microaggregates (yellow stars). Under polarized light the direction of birefringence is positive according to the long axis of HA crystals, like that of collagen fibers. **(A)** HA crystals, unstained sections, viewed under polarized light, ×100, **(B)** ×200, **(C)** ×100, **(D)** ×200.

Figures 4A–D demonstrates characteristic HA crystal deposits in a patient with clinically diagnosed primary synovial chondromatosis.

### Comparative histological analyses of apatite rheumatism, chondrocalcinosis and primary synovial chondromatosis

Apatite rheumatism, chondrocalcinosis and primary synovial chondromatosis were equally characterized by the accumulation of HA and CPPD crystals in various tissues of different joints (with or without amorphous mineral deposition, and with or without chondroid, osteoid and/or bone formation).

Forty-three (43) joints (knee 25, hip 9, shoulder 4, elbow 3 and wrist 2) of 37 patients were operated; 93 tissue samples of 37 patients were available and were examined with the light microscope.

The surgeries of patients with clinically diagnosed apatite rheumatism, chondrocalcinosis and primary synovial chondromatosis are summarized in Table 3.

**TABLE 3:** Forty-three operated joints of 37 patients with available 93 tissue samples.

Cl Dx/Patients	Sample	Knee	Hip	Shoulder	Elbow	Wrist	Surgery
AR *n* = 4	16	5	1	3	0	0	9
Ch-C *n* = 16	40	8	4	1	1	2	16
prSynCh *n* = 17	37	12	4	0	2	0	18
Total *n* = 37	93	25	9	4	3	2	43
In % of total		58.14	20.93	9.30	6.98	4.65	100.0

Tissue samples of 3 patients with clinically diagnosed prSynCh were not available; Table 3 summarizes only the 18 operated joints of 17 patient with 37 tissue samples.

The most common indication for surgery was registered in the case of the knee (58.14%), followed by the hip (20.93%), shoulder (9.30%), elbow (6.98%), and wrist (4.65%).

AR, Apatite rheumatism; Ch-C, Chondrocalcinosis; prSynCh, primary synovialis chondromatosis; Cl Dx, Clinical Diagnosis; Pts, Patients; Ts, Tissue sample.

Massive amorphous calcium phosphate [Ca_3_(PO_4_)_2_] and/or calcium carbonate [CaCO_3_] deposition characterized apatite rheumatism and chondrocalcinosis, chondroid formation was minimal (osteoid and/or bone formation was not detected).

Abundant chondroid, osteoid and/or bone formation was characteristic of primary synovial chondromatosis; amorphous mineral deposition occurred occasionally and was minimal.

The average mineral deposits, chondroid, osteoid and/or bone formation in tissue sections of patients with clinically diagnosed apatite rheumatism, chondrocalcinosis and primary synovial chondromatosis are summarized in Table 4.

**TABLE 4:** Amorphous mineral deposits, chondroid, osteoid and/or bone formation in tissue samples of patients with clinically diagnosed AR, Ch-C, and prSynCh.

Clinical diagnosis patients	Tissue samples	Calcification	Calcification/Tissue samples	Chondroid and/or bone formation	Chondroid and/or bone formation/Tissue samples
AR *n* = 4	16	17	1.063	1	0.063
Ch-C *n* = 16	37	39	1.054	2	0.054
prSynCh *n* = 17	37	10	0.270	79	2.135
Total of patients *n* = 37	90	66	0.733	82	0.911

The amount of calcium phosphate [Ca_3_(PO_4_)_2_] and/or calcium carbonate [CaCO_3_], furthermore chondroid, osteoid and/or bone formation was determined in 37 of 40 tissue samples with chondrocalcinosis; three tissue samples of one patient with clinically diagnosed Ch-C were not available for histological evaluation (tissue samples were too small).

In prSynCh the mineral deposits, chondroid, osteoid and/or bone formation were analyzed in 37 tissue samples of 17 patients.

Seventy-three (73) unstained tissue sections of 28 patients were examined with polarized light.

In unstained tissue sections of various tissues of joints HA and CPPD crystals existed side by side with different prevalence.

HA crystals were detected in 48, and CPPD in 43 cases of 73 unstained tissue sections.

HA crystals were observed without CPPD crystals in some cases, but usually were accompanied with some CPPD crystals.

CPPD crystals were not found without HA crystals (using Red I compensator the HA crystals with weak birefringence were not always detected because of the reduced illumination).

Table 5 summarizes the prevalence of HA and CPPD crystals in various tissue structures of different joints of 28 patients with the clinical diagnosis of apatite rheumatism, chondrocalcinosis and primary synovial chondromatosis.

**TABLE 5:** Distribution of HA and CPPD crystals in various tissue structures of different joints in unstained sections (*n* = 73) of patients with clinically diagnosed AR, Ch-C, and prSynCh.

Clinical diagnosis	Synovial membrane	Capsule	Bome/Cart	Bursa	Tendon	∑ of Ts
Apatite rheumatism						
Number of tissue samples	7	5	2	2	0	16
HA positivity	7	1	0	2	0	10
in %	100	20	0	100	0	62.50
CPPD positivity	7	0	0	2	0	9
in %	100	0	0	100	0	56.25
Chondrocalcinosis						
N of tissue samples	15	15	5	3	2	40
HA positivity	15	7	2	1	1	26
in %	100	33	0	33	50	65.00
CPPD positivity	15	5	0	1	1	22
in %	18	18	1	0	0	55.00
prSynCh						
Number of tissue samples	8	8	1	0	0	17
HA positivity	8	4	0	0	0	12
in %	100	50	0	zero divisor	zero divisor	70.59
CPPD positivity	8	4	0	0	0	12
in %	100	50	0	zero divisor	zero divisor	70.59
Total number of Ts	30	28	8	5	2	73
In % of ∑ of Ts	41.10	38.36	10.96	6.85	2.74	100.00

The prevalence of HA and/or CPPD was determined with polarized light in 73 unstained tissue sections of 28 patients.

prSynCh, primary synovialis chondromatosis; Ts, Tissue sample.

Prevalence of HA and CPPD crystals was detected in 93 HE stained tissue sections of 37 patients.

In contrast to the HE stained sections the prevalence of HA and CPPD crystals was detected only in 73 unstained tissue sections of 28 patients; 20 tissue samples of 9 patients with prSynCh were not available for analysis.

Demonstration of HA and CPPD crystals was more effectively expressed in percent of positive cases by the non-staining technique according to Bély and Apáthy (2013) than with conventional HE stains.

Table 6 summarizes the occurrence of HA and CPPD crystals in 93 HE stained tissue sections of 37 patients with the clinical diagnosis of AR, Ch-C, and prSynCh, compared to the 73 unstained tissue sections of 28 patients.

**TABLE 6:** Distribution of HA and CPPD crystals in tissue sections stained with HE (*n* = 93) compared to unstained sections (*n* = 73).

Staining technics clinical diagnosis	HE stained tissue sections prevalence *n* (%)		Unstained tissue sections prevalence *n* (%)	
	HA	CPPD	HA	CPPD
Apatite rheumatism				
Patients *n* = 4	0 (0% of 4)	3 (75.0% of 4)	4 (100% of 4)	4 (100% of 4)
Tissue samples *n* = 16	0 (0% of 16)	3 (18.75% of 16)	10 (62.5% of 16)	9 (56.25% of 16)
Chondrocalcinosis				
Patients *n* = 16	0 (0% of 16)	5 (31.25% of 16)	16 (100% of 16)	16 (100% of 16)
Tissue samples *n* = 40	0 (0% of 40)	8 (20.0% of 40)	26 (65% of 40)	22 (55% of 40)
Primary synovial chondromatosis				
Patients *n* = 17 of 20^a^	0 (0% of 17)	4 (23.53% of 17	8 (100% of 8)	8 (100% of 8)
Tissue samples *n* = 37 of 17 Pts^b^	0 (0% of 37)	4 (10.81% of 37)	12 (70.59% of 17)	12 (70.59% of 17)
Total n of patients *n* = 37^a^	0 (0% of 37)	12 (32.43% of 37)	*n* = 28 (73.68% of 38)	*n* = 28 (100% of 38)
Total n of tissue sections *n* = 93^b^	0 (0% of 93)	18 (19.35% of 93)	*n* = 48 (65.75% of 73)	*n* = 43 (58.90% of 73)

HA or CPPD crystal fragments in traces with uncertain birefringence were regarded negative.

^a^ Tissue samples of 3 patients with clinically diagnosed prSynCh were not available therefore only 17 of 20 patients were taken into account.

^b^ Prevalence of HA and CPPD crystals was determined in 37 HE stained tissue sections of 17 patients, and in 17 unstained tissue sections of 8 patients with prSynCh; 20 tissue sections of 9 patients with prSynCh were not analyzed with the non-staining technique.

In HE stained tissue sections of MSU, AR, Ch-C or prSynCh cholesterol crystals (CC—[C_27_H_46_O]), and/or crystalline liquid lipid droplets (CL) were not found.

Variable amounts of cholesterol (CC—[C_27_H_46_O]), and crystalline liquid lipid droplets (CL) were demonstrated with Bély and Apáthy’s non-staining technique (2013) in 3 of 4 with AR, in 11 of 16 with Ch-C, and in 2 of 8 patients with clinical diagnosis of prSynCh.

**FIGURE 5:**
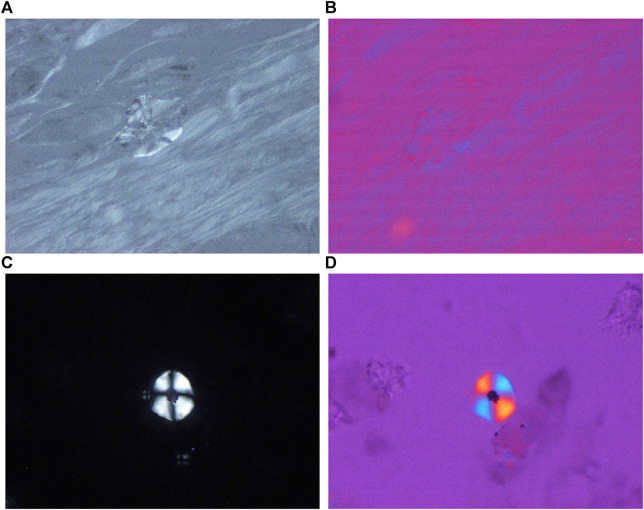
Apatite rheumatism, synovial membrane, semiliquid cholesterol crystals; unstained tissue section, viewed by polarized light, with and without Red 1 compensator. The birefringence of semiliquid cholesterol and lipid liquid crystals is identical with the birefringence of collagen fibers, and is positive in **(A,B)**. **(A)** Semiliquid cholesterol crystal, unstained sections, viewed under polarized light without Red I. compensator, ×200, **(B)** with Red 1 compensator, same as **(A)** ×200, **(C)** Lipid liquid crystals, stained for cholesterol according to Schultz, viewed under polarized light, without Red I. compensator, ×600, **(D)** with Red 1 compensator, same as **(C)** ×600.

Figures 5A–D demonstrates characteristic semiliquid cholesterol and crystalline liquid lipid droplets.

In unstained sections of 5 patients the typical HA and CPPD crystals were accompanied with crystals of different shapes, sizes, and arrangements like a hair braid or reminiscent of a shingle roof.

Crystals with such different shapes, sizes and arrangements occurred in all three patient groups; in 1 patient with the clinical diagnosis of AR, in 2 with Ch-C, and in further 2 patients with prSynCh.

The birefringence of these crystals was stronger than that of HA crystals, and weaker than that of CPPD. The birefringence of the closely fitting parallel crystals was positive and negative in the same axis position.

**FIGURE 6:**
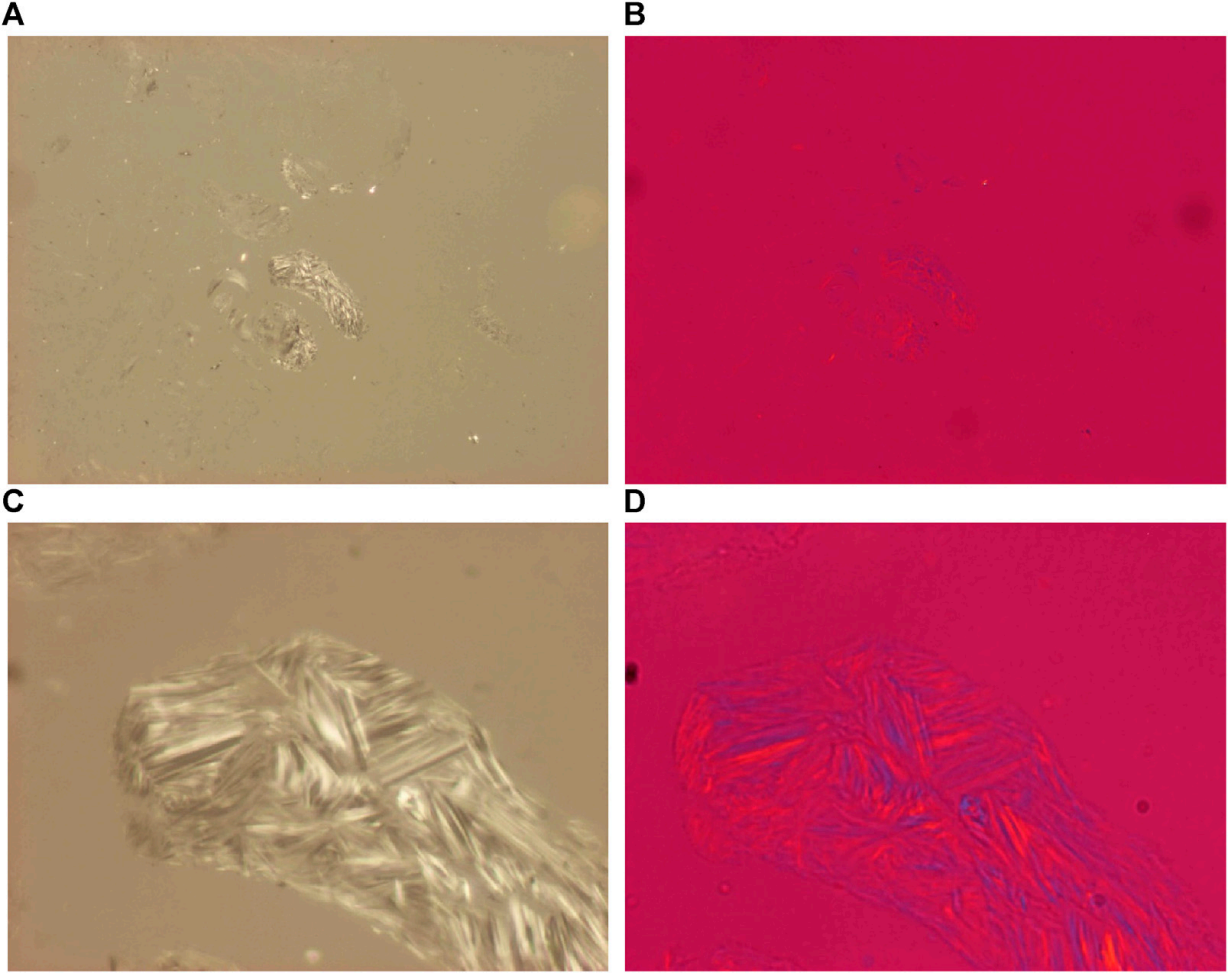
Apatite rheumatism, knee joint synovial membrane, unidentified rod-shaped crystals in a hair braid (vortex) like arrangement. The shape, size and arrangement of these tightly fitting unidentified crystals differs from that of HA and/or CPPD crystals. The birefringence of these crystals is positive and negative parallel to the long axis of the crystals. The rod shape of the crystals is supported by the birefringence in the dark-light band of the same width. **(A)** HA crystals, unstained sections, viewed under polarized light, ×100, **(C)** ×600. **(B)** HA crystals, unstained sections, viewed under polarized light, using Red I compensator [same field as **(A)**, ×100, **(D)** ×600].

Figure 6 demonstrates rod-shaped crystals of different sizes and arrangement than that of HA or CPPD crystals.

## Discussion

Apatite rheumatism (apatite rheumatism, apatite rheumatism, hydroxyapatite arthritis, calcifying tenosynovitis, Milwaukee syndrome, frozen shoulder, calcific tendinitis) is a HA crystal induced arthropathy, while chondrocalcinosis [pseudogout, calcific gout, polyarticular (familial) chondrocalcinosis, pyrophosphate arthropathy] is thought to be a CPPD crystal induced arthropathy.

These metabolic diseases are regarded as different clinical entities [26–37], although the clinical symptoms are the same, the most frequently affected joints overlap, and there is currently no difference in their treatment [37–40].

Bywaters (1972) reported coexistent CPPD and HA crystal deposits in the synovial membrane in rheumatoid arthritis without suspicion of a single metabolic disease [41].

Hayes and Conway (1990) described co-occurrence of CPPD and HA crystals in periarticular soft tissues, especially in tendons; the co-occurrence of HA and CPPD were regarded as a mixed metabolic malady of apatite rheumatism and calcium phosphate crystal deposition disease [42] quoting Resnick [43].

Using the Bély and Apáthy non-staining technique (2013) varying amounts of HA and CPPD crystals were observed side by side in apatite rheumatism and in chondrocalcinosis [24, 25].

These studies indicate that apatite rheumatism and chondrocalcinosis are HA and CPPD induced maladies, of the same basic metabolic disorder [24, 25].

The classic clinical diagnosis should be based on the presence of the dominant crystals in the deposits and not on the clinical symptoms or their localization.

Primary synovial chondromatosis (Reichel disease) of unknown origin is considered to be a distinct clinical entity [27, 30, 44, 45].

The constant presence of HA and CPPD crystals demonstrated with the unstained technique in primary synovial chondromatosis suggests that prSynCh and apatite rheumatism or chondrocalcinosis are related, but distinct metabolic diseases, which belong to the same group of metabolic maladies.

HA and CPPD crystals in apatite rheumatism or chondrocalcinosis were accompanied in most cases with abundant calcium phosphate [Ca_3_(PO_4_)_2_] and/or calcium carbonate [CaCO_3_] deposits, while chondroid and/or osteoid formation is rare and minimal (Table 5).

The presence of HA and CPPD crystals in prSynCh was characterized by conspicuous chondroid and/or bone formation in contrast to AR or Ch-C, while the amorphous calcium phosphate [Ca_3_(PO_4_)_2_] and/or calcium carbonate [CaCO_3_] deposition was rare and minimal (Table 5).

An inflammatory reaction was inhibited or moderated in the presence of amorphous minerals in AR or Ch-C, and inhibited or moderated in the presence of chondroid, osteoid and/or bone formation in prSynCh.

We consider amorphous calcium deposition a first-line response, and chondroid and/or osteoid formation a second-line defense mechanism against the crystals, reducing or moderating inflammation.

In our view the prSynCh is a defective variant of HA and CPPD induced metabolic disorders, where the deficient mineralization is replaced by chondroid and/or bone formation. These abilities may be determined by genetic or other, unidentified factors, respectively influenced by intrinsic and/or extrinsic causes.

The relatively high mean age of patients with clinically diagnosed AR (74.00 years) at the time of surgery compared with the relatively lower mean age of patients with clinically diagnosed Ch-C (63.67 years) suggests that the small, more soluble, phagocytosed HA crystals are more tolerable for a longer period of time, and provoke lesser clinical symptoms than the larger, less soluble, more irritative CPPD crystal deposits.

There was no significant difference in mean age between patient cohorts with AR and Ch-C (*p* < 0.844) supporting the possibility that AR and Ch-C represent the same metabolic disorder.

The mean age of the patients with the clinical diagnosis of prSynCh was low in comparison with AR or Ch-C, probably caused by the synovial calcified and/or ossified loose bodies.

Crystal induced arthropathies are progressive maladies.

All diseases characterized by progressive deposition of crystals or proteins (amyloid), fat, etc., begin in organs and on tissue structures that are frequently involved by marked deposits; deposition starts later where deposits are infrequent or less marked.

In connection with systemic amyloidosis this general rule was analyzed in detail and confirmed in our previous studies [46–49].

According to the Wald sequence analysis [50], the “most common deposit is the earliest deposit.”

A disease with progressive cumulative depositions probably starts where small early deposits are the most common.

This is a fundamental rule for all progressive cumulative disorders, including crystal induced arthropathies.

In patients with clinically diagnosed AR, Ch-C, and prSynCh the most commonly involved joint was the knee 25 (58.14% of 43 surgeries), followed by the hip 9 (20.93%), shoulder 4 (9.30%), elbow 3 (6.98%), and wrist 2 (4.65%) (Table 4).

Regarding the tissue structures of the joints, the most commonly involved tissues were the synovial membranes (41.10%), followed by the capsules (38.36%), bone and/or cartilage (10.96%), bursae (6.85%), and tendons (2.74%) (Table 6).

These data support the theory that *crystal deposition begins in all three metabolic maladies in the synovial membrane of the knee, succeeded by other structures and other joints*.

Unstained sections of 73 surgical samples of 37 patients showed more frequent, and more marked HA crystal deposition than CPPD crystal deposition; HA crystals were present in 48 (65.75%), while CPPD only in 43 (58.90%) of 73 tissue samples (Table 6).

In our patients with clinically diagnosed AR, Ch-C or prSynCh the crystal deposition appears to have begun with HA crystal deposition, followed by CPPD deposition.

This histologically outlined progressive process is also reflected in the clinically recognized stages of crystal-induced diseases.

These were recommended for chondrocalcinosis: asymptomatic CPP disease (“asymptomatic CPPD”), acute CPP crystal arthritis (“pseudogout”), and chronic CPP crystal inflammatory arthritis (“pseudo-RA”) [37], and for apatite rheumatism: pre-calcific, formative, resting phases, including resorptive phase characterized by inflammation, and post-calcific phase characterized by reparative processes [51].

## Conclusion

The non-staining technique of Bély and Apáthy is a much more effective method for the demonstration of crystals in metabolic diseases than the conventional stains and reactions.

Apatite rheumatism, chondrocalcinosis, and primary synovial chondromatosis are related metabolic disorders, provoked by HA and CPPD depositions.

AR and Ch-C are different stages of the same metabolic disorder, which differ from the prSynCh in amorphous mineral production, furthermore in the production of chondroid, osteoid and/or bone in prSynCh.

The authors assume that the prSynCh is a defective variant of HA and CPPD induced metabolic disorders with reduced mineralization capabilities, where the deficient mineralization is replaced by chondroid and/or bone formation.

## Data Availability

All relevant data is contained within the article and supplementary material. The original clinical and histological documents were filed in the authors’ department, but due to reconstruction of the institute are no longer available. Any further inquiries can be directed to corresponding author.

## Abbreviations

AR, Apatite rheumatism; Ch-C, Chondrocalcinosis; prSynCh, primary synovial chondromatosis; HA, Calcium hydroxyapatite—[Ca_5_(PO_4_)_3_(OH)]; CPPD, Calcium pyrophosphate dihydrate—[Ca_2_P_2_O_7_.2H_2_O]; CC, Cholesterol crystals—[C_27_H_46_O]; CL, Crystalline liquid lipid spherules; HE, Hematoxylin Eosin; Ts, Tissue samples; Pr. n^0^/y, Protocol number/year.

## AppendixBély and Apáthy’s “non-staining” technique

1. Tissue blocks of surgically removed specimens are fixed in 8% neutral buffered formalin (at pH 7.6 for >24 h at 20°C room temperature) [1, 12–16].
2. Tissue blocks are dehydrated in ethyl alcohol, and are embedded in paraffin using acetone as well as xylene—5 µm sections are cut.
3. Prolonged deparaffinization (3–5 days) in a thermostat at 56°C (daily changing xylene)
4. Chloroform–methanol I. (1:1) solution for 1 h
5. Chloroform–methanol II. (1:1) solution for 1 h or overnight
6. Dehydration in ethyl alcohol (two changes of 96% alcohol I-II. 30–30 min), and using terpene xylene, as well as xylene, mounting in Canada balsam, cover slip.

### Results

In deparaffinized tissue sections of formaldehyde fixed and paraffin embedded surgical specimens—without staining with aqueous dyes—the cholesterol crystals and crystalline lipids, furthermore the HA crystals are preserved, and are well detectable with polarized light.

In unstained sections MSU and CPPD crystals are more abundant than in sections stained with HE or with other staining.

## References

[1] Bély M Apáthy Á. Mönckeberg sclerosis – kristály indukálta angiopathia (Mönckeberg‟s sclerosis: crystal-induced angiopathy). Orvosi Hetilap (2013) 154(23):908–13. 10.1556/OH.2013.29628 23728314

[2] Bély M Apáthy Á. Vascular calcification and crystal deposition diseases - a comparative study on 67 amputated legs with atherosclerosis and/or Mönckeberg sclerosis. EC Cardiol (2021) 8(10):01–19.

[3] Pearse AGE. Fat soluble colorant methods. In: Pearse AGE, editor. Histochemistry theoretical and applied. Volume one: p. 129-131, Volume two: analytical technology, Lipids, lipoproteins and proteolipids. Ch. 16, p. 786-849. Edinburgh, London, Melbourne and New York: Churchill Livingstone (1985).

[4] Swan A Chapman B Heap P Dieppe P. Submicroscopic crystals in osteoarthritic synovial fluids. Ann Rheum Dis (1994) 53(7):467–70. 10.1136/ard.53.7.467 7944620 PMC1005372

[5] Pay S Terkeltaub R. Calcium pyrophosphate dihydrate and hydroxyapatite crystal deposition in the joint: new developments relevant to the clinician. Curr Rheumatol Rep (2003) 5(3):235–43. 10.1007/s11926-003-0073-x 12744817

[6] Shidham V Chivukula M Basir Z Shidham G. Evaluation of crystals in formalin-fixed, paraffin-embedded tissue sections for the differential diagnosis of pseudogout, gout, and tumoral calcinosis. Mod Pathol (2001) 14(8):806–10. 10.1038/modpathol.3880394 11504841

[7] Forster CJ Oglesby RJ Szkutnik AJ Roberts JR. Positive alizarin red clumps in Milwaukee shoulder syndrome. J Rheumatol (2009) 36(12):2853. 10.3899/jrheum.090163 19966203

[8] Gatter RA Schumacher HR. “Microscopic findings under compensated polarized light and phase light” in: gatter RA. In: Schumacher HR, editor. A practical handbook of joint synovial fluid analysis. Philadelphia, London: Lea & Febiger (1991). p. 46.

[9] Paul H Reginato AJ Schumacher HR. Alizarin red S staining as a screening test to detect calcium compounds in synovial fluid. Arthritis Rheum (1983) 26(2):191–200. PMID: 6186260. 10.1002/art.1780260211 6186260

[10] Shoji K. Alizarin red S staining of calcium compound crystals in synovial fluid. Nihon Seikeigeka Gakkai Zasshi (1993) 67(4):201–10. PMID: 7686572. 7686572

[11] Yang JH Oh KJ Pandher DS. Hydroxyapatite crystal deposition causing rapidly destructive arthropathy of the hip joint. Indian J Orthopaedics (2011) 45:569–72. 10.4103/0019-5413.87139 PMC3227365 22144754

[12] Bély M Apáthy Á. A simple method of diagnostic pathology for identification of crystal deposits in metabolic and crystal induced diseases (2022). Available from: http://structural-crystallography.imedpub.com/archive.php (Accessed February 15, 2016).

[13] Bély M Apáthy A. Metabolic diseases and crystal induced arthropathies technic of non-staining histologic sections - a comparative study of standard stains and histochemical reactions. Clin Arch Bone Jt Dis (2018) 1(2). 10.23937/cabjd-2017/1710007

[14] Bély M Apáthy A. Crystal deposits in tissue of patients with chondrocalcinosis and apatite rheumatism – microscopic identification of CPPD and HA with the non-staining technique of Bely and Apáthy. BAOJ Clin Trials (2018) 4(1):018.

[15] Apáthy Á Bély M. AB1262 amorphous mineralization, chondroid and/or osteoid formation in crystal induced metabolic disorders – a comparative study of apatite rheumatism, chondrocalcinosis and primary synovial chondromatosis apatite rheumatism, chondrocalcinosis, primary synovial chondromatosis. Ann Rheum Dis (2023) 82(1):1857–8. 10.1136/annrheumdis-2023-eular.193

[16] Bély M Apáthy Ά. A comparative microscopic study of apatite rheumatism, chondrocalcinosis and synovial chondromatosis – HA and CPPD induced metabolic disorders. EC Pulmonology Respir Med (2023) 12(6):01–17.

[17] Ohira T Ishikawa K. Preservation of calcium pyrophosphate dihydrate crystals: effect of Mayer's haematoxylin staining period. Ann Rheum Dis (2001) 60(1):80–2. PMID 11114290. 10.1136/ard.60.1.80 11114290 PMC1753352

[18] Carson FL. “Mayer’s hematoxylin” In: carson FL. In: Histotechnology. Chicago: ASCP Press (1990). p. 100–5.

[19] McManus JFA Mowry RW. Methods of general utility for the routine study of tissues, sodium Alizarin sulfonate stain for calcium and Von Kossa’s method for phosphates and carbonates. In: McManus JFA Mowry RW, editors. Staining methods, histologic and histochemical. New York: Hoeber PB Inc (1960). p. 55–72.

[20] Vacca LL. Alizarin red S. In: Vacca LL, editor. Laboratory manual of histochemistry. New York: Raven Press (1985). p. 333–4.

[21] Lillie RD. Von Kóssa’s method. In: Lillie RD, editor. Histpathologic technic and practical histochemistry. New York, Toronto, London: The Blakiston Division McGraw-Hill Book Company (1954). p. 264–5.

[22] Lentner C. “Statistical methods” volume 2. In: Lentner C Diem K Seldrup J, editors. Geigy scientific tables. Basle, Switzerland: Ciba-Geigy Limited (1982). p. 227.

[23] Bély M Apáthy A. Metabolikus megbetegedések – a kristályok csodálatos világa (Metabolic disorders – the wonderful world of crystals). Orvostovábbképző Szemle (2023) 9:66–81.

[24] Bély M Apáthy A. Crystal deposits in apatite rheumatism and chondrocalcinosis – microscopic identification of hydroxyapatite and calcium pyrophosphate dihydrate crystals with standard stains and histochemical reactions and with the nonstaining technique of Bély and Apáthy. EC Pulmonology (2022) 1:03–24.

[25] Bély M Apáthy Á. Apatite rheumatism and chondrocalcinosis are different stages of the same metabolic disorder – a clinicopathologic study of 21 patients with clinically diagnosed apatite rheumatism or chondrocalcinosis. J Interdiscip Histopathology (2022) 10:1–14.

[26] Reginato AM Yuvienco C. Hydroxyapatite crystal-induced rheumatology (2022). Available from: https://www.rheumatologyadvisor.com/home/decision-support-in-medicine/rheumatology/hydroxyapatite-crystal-induced/ (Accessed November 15, 2022).

[27] Mohr W. Kalziumpirophosphat-arthropathie, apatitkrankheiten, primäre synoviale osteochondromatose. In: Mohr W, editor. Gelenkpathologie, historische Grundlagen, Ursachen und Entwicklungen von Gelenkleiden und ihre Pathomorphologie. Berlin, Heidelberg: Springer-Verlag (2000). p. 193–212.

[28] Reginato AJ Reginato AM. Diseases associated with deposition of calcium pyrophosphate or hydroxyapatite. In: Ruddy S Harris ED Sledge CB, editors. Crystal-associated synovitis, section XV, kelly’s textbook of rheumatology. 6th ed. Philadelphia, London, New York, St. Louis, Sydney, Toronto: WB Saunders Company: A division of Harcourt Brace & Company (2001). p. 1377–90. Ch. 90.

[29] Žitňan D Sitaj Š. Chondrocalcinosis articularis Section L clinical and radiological study. Ann Rheum Dis (1963) 22:142–52. 10.1136/ard.22.3.142 14003799 PMC1007340

[30] Gardner DL McClure J. Metabolic, nutritional and endocrine diseases of connective tissue, synovial osteochondromatosis. In: Arnold E, editor. Pathological basis of the connective tissue diseases. 1st ed. London, Melbourne, Auckland: Great Britain (1992). p. 984–6. Ch. 24.

[31] McCarty DJ Lehr RJ Halverson PB. Crystal populations in human synovial fluid. Identification of apatite, octacalcium phosphate, and tricalcium phosphate. Arthritis Rheum (1983) 26:1220–4. 10.1002/art.1780261008 6626280

[32] Fassbender HG. Crystal-associated arthropathies” in Pathology and pathobiology of rheumatic diseases. 2nd ed. Berlin, Heidelberg, New York, Germany: Springer-Verlag (2002). p. 353–69. ch. 17.

[33] Gupta SJ. Crystal induced arthritis: an overview. INJR (Indian Journal of Rheumatology - formerly). J Indian Rheumatol Assoc (2002) 10:5–13.

[34] Rosenthal AK Ryan LM. Calcium pyrophosphate deposition disease. New Engl J Med (2016) 374:2575–84. 10.1056/NEJMra1511117 27355536 PMC6240444

[35] Dieppe PA Crocker P Huskisson EC Willoughby DA. Apatite deposition disease. A new arthropathy. Lancet (1976) 307:266–9. 10.1016/S0140-6736(76)91400-8 55584

[36] Dieppe PA. Milwaukee shoulder. Br Med J (Clin Res Ed). (1981) 283(6305):1488–9. PMC1507904. 10.1136/bmj.283.6305.1488 PMC1507904 6799034

[37] Rosenthal A Dalbeth N Romain PL. Clinical manifestations and diagnosis of calcium pyrophosphate crystal deposition (CPPD) disease”. Wolters Kluwer Canada (2024). Available from: https://www.uptodate.com/contents/clinical-manifestations-and-diagnosis-of-calcium-pyrophosphate-crystal-deposition-cppd-disease (Accessed November 01, 2023).

[38] Bachmann D Resnick D. “Calcium pyrophosphate dihydrate crystal deposition disease” and “Calcium hydroxyapatite crystal deposition disease”. In: Bachmann D Resnick D, editors. Radiological atlas of rheumatological diseases. Basel, Switzerland: Hoffmann-La Roche Ltd. (1994). p. 108–23.

[39] mayoclinic. Pseudogout (2022). Available from: https://www.mayoclinic.org/diseases-conditions/pseudogout/symptoms-causes/syc-20376983 (Accessed July 28, 2022).

[40] Pálinkás M Poór G. Kristályatrthritisek. In: Szekanecz Z Nagy G, editors. Reumatológia. Budapest: Medicina könyvkiadó Zrt (2019). p. 572. Ch. 41.

[41] Bywaters EGL. Calcium pyrophosphate deposits in synovial membrane. Ann Rheum Dis (1972) 31(3):219–21. 10.1136/ard.31.3.219-b PMC1005904 4338107

[42] Hayes CW Conway WF. Calcium hydroxyapatite deposition disease. RadioGraphics (1990) 10:1031–48. 10.1148/radiographics.10.6.2175444 2175444

[43] Resnick D. Calcium hydroxyapatite crystal deposition disease. In: Resnick D Niawayama G, editors. Diagnosis of bone and joint disorders. 2nd ed. Saunders Philadelphia (1988). p. 1733–64.

[44] GARD. Synovial chondromatosis – gard (2023). Available from: https://rarediseases.info.nih.gov/diseases/6054/synovial-chondromatosis (Accessed February, 2023).

[45] AAOS. Synovial chondromatosis - OrthoInfo – AAOS (2022). Available from: https://orthoinfo.aaos.org/en/diseases--conditions/synovial-chondromatosis (Accessed January 2022).

[46] Bely M Apathy A Pinter T Ratko J. Generalized secondary amyloidosis in rheumatoid arthritis. Acta morphologica Hungarica (1992) 40:49–69. 1365773

[47] Bély M. “Identification of amyloid deposits by histochemical methods of romhányi” in: bély M, guest editor. Amyloid (2001) 8.2:177–82.

[48] Bély M Apáthy Á. Clinical pathology of rheumatoid arthritis: cause of death, lethal complications and associated diseases in rheumatoid arthritis. Budapest: Akadémiai Kiadó (2012). p. 1–440.

[49] Bély M Apáthy Á. Formal pathogenesis of systemic and localized amyloidosis. EC Cardiol (2019) 6(5):444–69.

[50] Wald A. Sequential analysis. New York: Wiley Mathematical Statistics Series, Chapman & Hall (1947).

[51] Uhthoff HK Loehr JW. Calcific tendinopathy of the rotator cuff: pathogenesis, diagnosis, and management. J Am Acad Orthopaedic Surgeons (1997) 5(4):183–91. 10.5435/00124635-199707000-00001 10797220

